# Roles of Mitochondrial DNA Mutations in Stem Cell Ageing

**DOI:** 10.3390/genes9040182

**Published:** 2018-03-27

**Authors:** Tianhong Su, Doug M. Turnbull, Laura C. Greaves

**Affiliations:** 1 Wellcome Centre for Mitochondrial Research, Institute of Neuroscience, Newcastle University, Newcastle upon Tyne NE2 4HH, UK; t.su2@newcastle.ac.uk (T.S.); doug.turnbull@newcastle.ac.uk (D.M.T.); 2 LLHW Centre for Aging and Vitality, Newcastle University Institute for Aging, The Medical School, Newcastle upon Tyne NE2 4HH, UK

**Keywords:** stem cell, mitochondrial DNA mutation, ageing, purifying selection, mitochondrial mutagenesis, progenitor, mitochondrial disease, ageing mouse model, mitochondrial DNA-mutator mouse, mitochondria

## Abstract

Mitochondrial DNA (mtDNA) mutations accumulate in somatic stem cells during ageing and cause mitochondrial dysfunction. In this review, we summarize the studies that link mtDNA mutations to stem cell ageing. We discuss the age-related behaviours of the somatic mtDNA mutations in stem cell populations and how they potentially contribute to stem cell ageing by altering mitochondrial properties in humans and in mtDNA-mutator mice. We also draw attention to the diverse fates of the mtDNA mutations with different origins during ageing, with potential selective pressures on the germline inherited but not the somatic mtDNA mutations.

## 1. Introduction

Ageing is a process where tissue gradually loses homeostasis and regeneration [1]. This process is systemic and closely associated to age-related changes in somatic stem cells [2]. These cells renew themselves and differentiate into tissue-specific daughter cells for tissue maintenance and regeneration. The age-related alterations in somatic stem cell properties include failure to generate functional progenies, depletion of the stem cell pool and cancerous transformation [3]. These changes largely affect mitotic tissue, such as blood, intestine and skin, where the stem cells actively produce progenies to maintain the high turnover of the tissue [4,5]. However, they also contribute to ageing post-mitotic tissue, such as brain and muscle, though stem cells in these tissues are considered quiescent under normal physiological conditions and activated in response to damage for repairing the tissue [6,7]. 

Mitochondria synthesize ATP via oxidative phosphorylation (OXPHOS) through five multi-subunit complexes. Mitochondria contain their own DNA (mtDNA), which encodes key subunits of these complexes. Replication of the mitochondrial genome is independent of the cell cycle [8]. In addition, mtDNA is susceptible to damage due to lack of histone protection and proximity to oxidative stress [9]. Due to these reasons, compared with the nuclear DNA, mtDNA is more prone to mutations. Multiple copies of mtDNA reside in a cell. Mutations of mtDNA usually occur as a proportion of the total copies and once they reach a threshold, mitochondria will display respiratory chain deficiency, a consequence of which is potentially excessive production of reactive oxygen species (ROS) [10]. mtDNA is maternally transmitted through germline with a bottleneck effect, where only a small portion of mtDNA molecules are distributed into each primordial germ cell, which are amplified in later oogenesis. As a result, mature oocytes may have very different levels of mtDNA mutations [11].

Ageing is accompanied by a reduction of mitochondrial function, resulting in respiratory chain defects which are thought to be associated with the accumulation of somatic mtDNA mutations [12]. The age-related change in mitochondria may in turn accelerate the ageing process [13]. Although the significance of mtDNA mutations in various parenchymal cells in normal ageing and age-related degenerative diseases has been broadly studied [14], the findings might not be able to be extrapolated to stem cells, as they are distinct from somatic cells in terms of biological and metabolic characteristics. With an increasing amount of research linking mtDNA mutations to stem cell ageing in the last decade, we discuss how the somatic mtDNA mutations behave during ageing in stem cell populations and how they potentially influence phenotypes of the stem cells with the evidence from the recent studies. Specifically, we want to draw attention to an intriguing age-related phenomenon of the germline inherited mtDNA mutations in patients with mitochondrial disorders, as opposed to the accumulated somatic mtDNA mutations in normal individuals, as mtDNA mutations from different origins seem to have diverse fates with age.

## 2. Somatic mtDNA Mutations in Normal Ageing Humans

**Figure 1:**
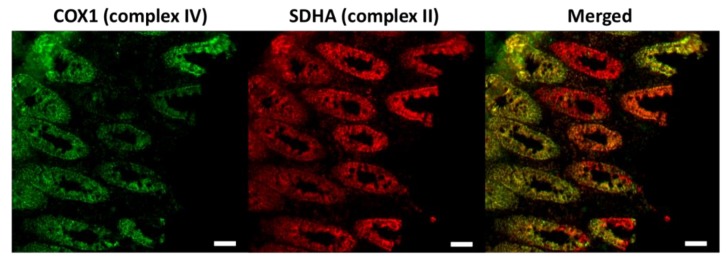
Immunofluorescence images of the respiratory chain deficiency caused by the age-dependent accumulation of somatic mitochondrial DNA (mtDNA) mutations in the colon. Respiratory chain complex IV (marked by the COX1 antibody) is encoded by both mtDNA and nuclear DNA (nDNA), which is affected by the increased burden of mtDNA mutations. Complex II (labelled by the SDHA antibody) is entirely encoded by nDNA. The complex IV deficient colonic cells are indicated red in the merged picture. COX1, a key subunit of complex IV encoded by mtDNA. SDHA, one of the four nuclear encoded subunits of complex II. Scale bar: 50 µm.

Respiratory chain defects have been observed in a variety of tissues in normal ageing humans, including the tissue traditionally considered post-mitotic, such as skeletal muscle [15], heart [16] and brain [17]; tissue with substantial mitotic potential after injury, for example, the liver [18]; as well as the typical mitotic tissue such as the epithelium of the stomach [19], small intestine [20] and colon (Figure 1) [21,22]. Various somatic mtDNA mutations were found to clonally expand and accumulate to high levels with age in the respiratory chain deficient areas of the tissue, regardless of their pathogenicity [21,23,24,25,26,27]. In addition, Shin et al. found increased mtDNA heterogeneity in CD34+ marked haematopoietic stem cell (HSC) and the progenitor cells both in the bone marrow and the peripheral blood from the adult donors compared to the homogenous umbilical cord blood [26,28]. In the intestinal crypt where all the cells derive from the stem cells located at the base of the crypt, the same somatic mtDNA mutation was determined at different regions along the OXPHOS defective crypt, indicating that they originated from a communal stem cell that harboured the mutation [20]. Monoclonal conversion where a single stem cell with the accumulated somatic mtDNA mutation passes the mutation to its progenies to occupy the entire crypt has also been observed in the stomach [19]. These studies provide robust evidence that in mitotic tissue, the accumulation of the somatic mtDNA mutation during ageing initiates in stem cells. However, whether the mutations aggregate in the stem cell of post-mitotic tissue, such as neural stem cells and satellite cells (muscle stem cell) and how they influence the fate of their progenies in aged humans remains unclear. 

The accumulation of the somatic mtDNA mutation during ageing can be entirely achieved by random genetic drift, which takes place early in life in contrast to the damage to mtDNA by excessive ROS production from the respiratory chain defect [29]. The pathogenicity of the acquired mtDNA mutations is markedly higher than in germline variants, suggesting that the purifying selective mechanism against germline mtDNA mutations is absent from the somatic mtDNA mutations [30].

The occurrence and the spread of the somatic mtDNA mutations during ageing are notably tissue-specific, potentially influenced by the mitotic capability of the tissue. In contrast to miscellaneous mtDNA point mutations found in the colonic stem cell populations, no large-scale deletions have been detected in any OXPHOS deficient crypts [21]. Post-mitotic brain and muscle preferentially accumulate mtDNA deletions instead of point mutations with age [31,32]. It has been proposed that neurons, which are highly energy consuming and sensitive to ROS, tend to acquire mtDNA deletions during the process of repairing the mtDNA that are injured by ROS [33]. Whereas in mitotic tissue, mtDNA mutations are likely to arise from errors during replication [31]. Furthermore, the stem cell niche is involved in dispersing the somatic mtDNA mutations specifically in mitotic tissue during ageing. Gland and crypt fission from the stem cell niche in the stomach and colon, respectively, have been shown to spread the mutation from a single unit to form a patch [19,34]. 

However, limited numbers of human studies delve into how somatic mtDNA mutations affect the function of stem cells and their progenies to contribute to the ageing phenotype of the tissue, and the findings are controversial. Respiratory chain deficiency due to accumulated somatic mtDNA mutations in colonic stem cells diminished the cell population of the crypt by weakening the cell proliferation and enhancing the apoptosis of the stem cell progenies [35]. In contrast, the level of the lineage markers labelling all the differentiated daughter cells derived from the OXPHOS deficient stem cell were shown to be normal when compared to OXPHOS normal epithelium of the gastric unit and the small intestinal crypt, indicating that the functionality of the progenies were not affected [19,20]. Additionally, the patch of hepatic progenitor cells and their descendants with OXPHOS defects did not show any synthetic, metabolic or proliferative impairment [18]. Due to restricted sources of human tissue samples, difficulties in tracing the stem cell dynamic condition in humans, and shortage of the stem cell markers, the mechanism by which somatic mtDNA mutations influence stem cell ageing still remains unknown in humans, and scientists have pursued animal models for investigation.

## 3. Ageing of Somatic Stem Cell Populations in mtDNA-Mutator Mice

The development of an mtDNA-mutator mouse model highlights the potentially important role of somatic mtDNA mutations in ageing. These mice carry a knock-in mutation in the exonuclease domain of the mtDNA polymerase γ (*Polg*), compromising the proofreading ability of *Polg*, which results in an accumulation of mtDNA mutations with age [36,37]. The mutant mice have a shortened lifespan and display a series of progeroid phenotypes, such as weight loss, kyphosis, hair greying and loss, impaired hearing, thin subcutaneous fat, osteoporosis, sarcopenia and sterility, which mimics the signs of normal human ageing [36,37]. In addition, the development of mitotic tissue including intestine, thymus and testicle, is largely affected in the mutant mice [37]. Mutator mice suffer progressive anaemia due to defective haematopoiesis, which is the main cause of death [36,37,38]. These findings provide evidence that links the accumulation of somatic mtDNA mutations to stem cell ageing.

### 3.1. Age-Dependent Dysfunction of Stem Cells and Their Progenitors in Mitotic Tissue

The age-dependent accumulation of the somatic mtDNA mutations in the mtDNA-mutator mice either alters the properties of stem cells per se or the downstream progenitors. Studies initially focused on the haematopoietic system, which is a well-established model for stem cell research. Reports show that the mice suffer from abnormal haematopoiesis for both erythroid and lymphoid lineage [38,39,40] and that this consequence caused by the mtDNA mutation in the haematopoietic component is intrinsic, as transplanting the bone marrow from the homozygous mutants to the wildtype mice recapitulates the mutant phenotype [38]. Different stages of the progenitor cells through haematopoiesis have been shown to be more perturbed than the HSCs. However, HSCs themselves show notably compromised ability for repopulation and self-renewal after serial transplantation [39]. In addition, studies show that the development of the haematopoietic system is dysregulated during embryogenesis in the mtDNA mutator mice [40]. Intestinal epithelium is another beneficial model for somatic stem cell research as it allows us to visualise the gradual cradle-to-grave manifestations of the stem cell progenies in a straightforward fashion. Research on the small intestinal epithelium of the mutant mice reveals abnormal cell proliferation and increased apoptosis in the Lieberkühn crypt, where intestinal stem cells (ISC) and transit-amplifying cells are located [41]. Furthermore, the organoids consisting of ISCs and Paneth cells derived from the isolated crypts of the mutants did not grow efficiently in vitro [41]. These alterations resulted in prolonged cell migration and swollen morphology of the intestinal epithelium, leading to impaired fat absorption of the mtDNA mutator mice [41]. Studies have shown that, as with humans, the mtDNA mutations accumulating in the colonic crypts of the heterozygous mutator mice randomly distribute through the mitochondrial genome with no advantageous selection towards pathogenic mutations [42]. The clonal expansion of the mutations can be simulated by the model of random genetic drift [42]. In contrast, there is a strong rapid negative selection against nonsynonymous mtDNA mutations in the protein-coding regions through germline transmission [11,43], underlining the difference in the mechanisms of the selection during ageing and germline transmission. 

### 3.2. Ageing of Progenitor Cells in Post-Mitotic Tissue

Compared to the severe symptoms due to mtDNA mutations in blood and other mitotic tissue, post-mitotic tissue seems less affected by mtDNA mutagenesis in the mtDNA mutator mice. Although respiratory chain function is preserved in the cerebrum, cerebellum and skeletal muscles, studies have shown direct alterations in the homeostasis of neural stem cells and muscle progenitors [40,44]. The number of the nestin-positive neuronal stem cells was decreased in the OXPHOS deficient subventricular zone of the adult mtDNA mutator mice, although the number of neurons was normal [40]. Neural stem cells (NSC) isolated from the embryos of the mutator mice showed strikingly reduced self-renewal ability, primarily because of the mtDNA mutagenesis, but not the secondary ROS, as these cells accumulated high levels of mtDNA point mutations, while barely showing respiratory chain deficiency [40]. Cardiac progenitor cells (CPC) isolated from the mtDNA mutator mice displayed attenuated proliferation and higher inclination to death [44]. Mutagenesis of mtDNA also blocked the metabolic transition of the mutant CPCs from glycolysis to OXPHOS as they differentiated, resulting in a massive cell death through differentiation [44]. Furthermore, myoblasts extracted from the mtDNA mutator mice were found to generate thinner myotubes in skeletal muscles [44]. These findings have underlined the functional change in somatic stem cells and precursors in the post-mitotic tissue due to age-related mtDNA mutagenesis. 

## 4. Potential Mechanism Whereby Age-Related mtDNA Mutagenesis Affects Somatic Stem Cells

**Figure 2:**
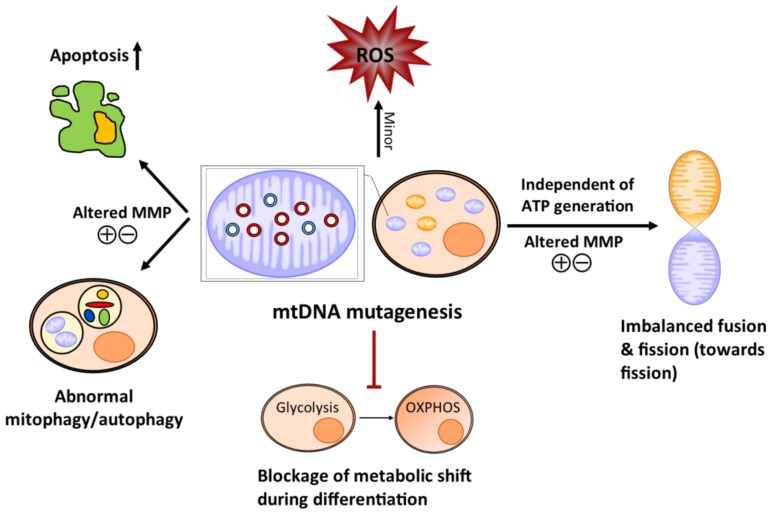
Schematic diagram of how the mitochondrial abnormalities caused by the age-dependent accumulation of somatic mtDNA mutations affect stem cell homeostasis in the mtDNA mutator mice. Mitochondrial DNA mutagenesis causes minor changes in reactive oxygen species (ROS)/redox level, which may alter stem cell identity (quiescence and regeneration). The somatic mtDNA mutation increases apoptosis and also shifts the level of mitophagy/autophagy in the stem cell population possibly through the loss of the mitochondrial membrane potential (MMP), which may eventually engender stem cell/progenitor depletion and accelerate stem cell senescence. Mitochondrial DNA mutagenesis imbalances mitochondrial dynamics towards fission independent of ATP production, which can affect stem cell self-renewal and differentiation. The amassing of somatic mtDNA mutations prevents stem cells from converting glycolysis to oxidative phosphorylation (OXPHOS) as they differentiate, resulting in cell death and failure to produce progenies. Normal mitochondria are coloured orange and dysfunctional mitochondria are in blue. Mutated mtDNA are red and the normal mtDNA are blue.

ROS is crucial for stem cell maintenance and is involved in manipulating stem cell differentiation, though too much ROS may be detrimental [45,46]. Mitochondrial DNA mutations are traditionally thought to affect the respiratory chain function of mitochondria, the main endogenous source of ROS, and interfere stem cell homeostasis [47]. The free radical theory of ageing based on mitochondria was initially proposed by Harman, where defective respiratory chain generates excessive ROS, damaging mtDNA and engendering somatic mtDNA mutagenesis with age, which further causes increased oxidative stress, forming a vicious cycle [48]. This theory was challenged when no obvious ROS elevation was found in different tissues of the mtDNA-mutator mice [37,49]. Analogous findings have been reported also in somatic stem cells [39]. However, treatment of antioxidant *N*-acetyl-l-cysteine (NAC) could rescue the impaired self-renewal ability of NSCs and abnormal haematopoiesis in the mutator mice [40]. The exact mechanism underlying these contradictory findings is as yet unknown. Despite an off-target possibility of NAC [50], these findings suggest that mtDNA mutagenesis may slightly alter the ROS/redox level (Figure 2), which is able to imbalance the quiescence and regeneration of the somatic stem cells [40]. This tiny change in the ROS/redox level might be insufficient to be detected, but it can be neutralized by NAC. However, the effect of the NAC on the stem cell population only seems to be effective if given during embryogenesis [40]. Of note, whether the effect of the antioxidant on the somatic stem cell benefits alleviating the ageing phenotype of either the stem cell or the tissue of the mutator mice remains controversial, as long-term treatment of NAC from embryogenesis did not rescue the aberrant erythropoiesis in the adult mutator mice [51]. In addition, calorie restriction, which reduces ROS and enhances antioxidative defence shows no effect on ameliorating the ageing phenotype of the mutator mice [52]. 

Recently, studies have reported another pre-ageing mouse model established by induced expression of mitochondrial-targeted endonuclease (Mito-Pstl) [53,54]. The main consequence of the double strand breaks of the mtDNA caused by the induction in these mice was mtDNA depletion [53,54]. In addition, mtDNA deletions accumulated in the post-mitotic brain and heart, but not in the lung and liver of these mice, though the expression of endonuclease was low in the brain and muscle but very high in the liver and lung [54]. Mitotic tissue was preferentially affected, with effects on the function of stem cells and progenitor cells. The differentiation of thymic progenitor cells was blocked at an early stage, leading to thymus shrinkage in these mice [54]. Satellite cells were lost in skeletal muscles of these mice, causing muscle wasting, but no change in the oxidative stress was detected in the muscle [53]. In addition, in the mitotic tissue that mainly displayed the ageing phenotype, no mtDNA depletion was found, implying that there might be another pathway that causes ageing rather than the secondary ROS generation caused by mtDNA depletion [54]. Together with the findings of the mtDNA-mutator mice, these observations suggest multi-factorial causes of ageing due to mtDNA mutagenesis or damage. In the cell model with the induction of the mtDNA endonuclease, elevated levels of ROS were detected, and this upregulated the ageing-related cell cycle arrest nuclear signalling pathway. This can be corrected by antioxidant NAC [54]. The absence of detectable changes in oxidative stress in either mtDNA-mutator mice or the Mito-Pstl mice is interesting, especially since many of the effects are reversed by NAC.

Accumulation of somatic mtDNA mutations can also cause dramatic alterations in mitochondrial dynamics, which is further able to perturb stem cell self-renewal and differentiation. During differentiation of the CPCs from the mtDNA mutator mouse, mitochondria displayed a series of abnormalities when compared to the WT mice, including imbalanced fusion and fission, reduced membrane potential, and poor development of the mitochondrial microstructure [44]. Mitochondrial dynamics can change mitochondrial morphology and regulate stem cell destiny by ROS signalling independent of generating ATP by OXPHOS [55]. Hence, though no change has been found in the ATP level in the stem cells from the mtDNA-mutator mice [39,41,44], the age-dependent accumulation of mtDNA mutations may still be able to alter the fate of stem cells secondarily through anomalous mitochondrial dynamics (Figure 2). 

In addition, the age-related mutagenesis of somatic mtDNA may hamper stem cell metabolism as they differentiate (Figure 2). Stem cells are naturally glycolytic, and actively suppress OXPHOS to maintain quiescence. Differentiation of stem cells requires a metabolic transition from glycolysis to OXPHOS to increase ATP production [45]. Somatic mtDNA mutations prohibited this metabolic shift as CPCs differentiate, leading to substantial cell death [44]. This could also be the reason for the blockage of the differentiation at different stages through the early erythropoiesis [39].

Furthermore, the quality control of mitochondria is perturbed in the stem cell population of the mtDNA-mutator mice, which may contribute to stem cell senescence. Autophagy is crucial for maintaining stem cell quiescence and stemness against ageing [56,57]. Studies have reported upregulated mitophagy rates in the CPCs [44] and inhibited autophagy in the early erythroid precursors of the mtDNA-mutator mice, the latter of which contributed to the malfunction of erythropoiesis [58]. Moreover, a lowered mitochondrial membrane potential (MMP) has been found in the HSCs, various haematopoietic progenitors, and the CPCs in the mtDNA-mutator mice [39,44], which is associated with mitophagy activation [59]. These observations indicate that mtDNA mutagenesis may affect stem cell function by interfering with the level of mitophagy/autophagy (Figure 2). However, how exactly mitophagy/autophagy responds to mtDNA mutagenesis in stem cells is uncertain, as the findings seem to be contradictory in different stem cell/progenitor populations. Of note, the loss of the MMP also occurs during apoptosis [60]. Consistently, strikingly increased apoptosis levels were found in the erythroid precursors and the ISC population (Figure 2) [39,41], which may subsequently lead to the depletion of the stem cell/progenitors. Surprisingly, no change in the apoptosis was observed in the HSC [39].

The effect of age-related mtDNA mutagenesis on somatic stem cell ageing seems highly tissue-specific. For haematopoiesis, it primarily affects the differentiation of downstream progenitor cells rather than HSCs themselves [39]. In contrast, it markedly affects NSCs but its impact fades during differentiation [40]. 

## 5. Behaviour of mtDNA Mutations Inherited through Germline with Age

mtDNA mutations from different origins seem to act differently with age, despite causing the same biochemical defect [14]. Mitochondrial disorders are caused by mtDNA mutations inherited through the germline or occurring during embryogenesis. As opposed to the acquired somatic mtDNA mutations that accumulate in stem cell populations with age, some of the inherited mtDNA mutations are lost in mitotic tissue over time. Pearson syndrome is a severe medical condition caused by a single, large-scale mtDNA deletion affecting the bone marrow haematopoietic compartment and causing pancytopenia in patients [61]. However, the anaemia and the vacuolated haematopoietic precursors in the bone marrow can be rescued by an age-related loss of the deletion and patients may subsequently develop Kearns-Sayer syndrome, which primarily affects post-mitotic tissue [62,63,64]. It has also been reported that the mutation level of the inherited m.3243A>G, the most common mtDNA point mutation that causes mitochondrial disease, decreased in blood and various epithelial tissues with age in patients and asymptomatic carriers [65,66,67,68]. The in silico simulation suggests that the loss of the mutation occurs in HSCs [69]. In addition, in a recently-developed mouse model of mitochondrial disease, the inherited m.5024C>T was lost in the blood of the mice with high mutation levels with age [70]. These studies imply that these inherited mtDNA mutations seem to be selected against in the stem cell population in contrast to the somatic mtDNA mutations, which are under no selective pressures [30]. However, no observations of the negative selection have been reported directly in stem cells in vivo, and the mechanism is as yet unexplored. Investigating the underlying mechanism of the mtDNA mutation with different origins reacting differentially to age is crucial for understanding both the process of normal ageing and development of the mitochondrial disease. 

## 6. Conclusions

We have provided evidence of the somatic mtDNA mutations accumulating in stem cell populations in normal humans and discussed their tissue-specific ability to expand clonally during ageing. The premature ageing mtDNA-mutator mouse model gives insight into how acquired mtDNA mutations affect the function of the stem cells and progenitors in both the mitotic and post-mitotic tissue, as well as the potential mechanisms by which age-related mtDNA mutagenesis affects stem cell homeostasis. We also highlight the intriguing contrast in the selective pressures against the mtDNA mutation with different origins in the fast-renewing mitotic tissue. Stem cells, which are responsible for tissue maintenance and regeneration, might be involved in the selective process against the inherited mtDNA mutation. Recently, studies have reported that stem cells might actively regulate their identity by manipulating the quality control of the mitochondria, for example, by removing the dysfunctional mitochondria [56] or by unevenly segregating young and aged mitochondria [71]. The quality control system might lose the function during ageing, leading to the absence of selective pressures on the somatic mtDNA mutations, which in turn accelerates ageing. We believe that investigating the mechanism of the difference in the selective pressure on the somatic and the inherited mtDNA mutations has a profound influence on understanding the progress of normal ageing, ageing-related degenerative diseases and cancer formation [72,73]. Studies have now provided several methods for preventing the germline transmission of the inherited mtDNA mutations to enable producing healthy children from affected mothers [74,75,76]. However, no efficacious interventions have been found for diseases caused by mutations that occur during embryogenesis. Understanding the mechanism of the selective pressure on the inborn mtDNA mutation will also propel the development of the treatment for mitochondrial disorders, especially for those occurring during embryogenesis and those that are recessive with late-onset.
